# Use of Tramadol in Pain Management of Neonates with Epidermolysis Bullosa: A Single-Center Experience

**DOI:** 10.3390/children13060745

**Published:** 2026-05-27

**Authors:** Jole Rechichi, Domenico Umberto De Rose, Flaminia Pugnaloni, Andrea Diociaiuti, Elisa Pisaneschi, Andrea Dotta, Annabella Braguglia, May El Hachem

**Affiliations:** 1 Neonatal Sub-Intensive Care Unit and Follow-Up, “Bambino Gesù” Children’s Hospital IRCCS, 00165 Rome, Italy; jole.rechichi@opbg.net (J.R.); annabella.braguglia@opbg.net (A.B.); 2 Neonatal Intensive Care Unit, “Bambino Gesù” Children’s Hospital IRCCS, 00165 Rome, Italy; flaminia.pugnaloni@opbg.net (F.P.); andrea.dotta@opbg.net (A.D.); 3 Dermatology Unit and Genodermatoses Research Unit, Translational Paediatrics and Clinical Genetics Research Division, “Bambino Gesù” Children’s Hospital IRCCS, 00165 Rome, Italy; andrea.diociaiuti@opbg.net (A.D.); may.elhachem@opbg.net (M.E.H.); 4 Translational Cytogenomics Research Unit, “Bambino Gesù” Children’s Hospital IRCCS, 00165 Rome, Italy; elisa.pisaneschi@opbg.net

**Keywords:** neonatal pain management, procedural pain, analgesia, NIPS, wound care

## Abstract

**Highlights:**

**What are the main findings?**

- Scheduled tramadol administration appeared feasible and was not associated with observed immediate hemodynamic or respiratory complications in this small cohort.
- No hemodynamic or respiratory adverse effects were observed during hospitalization or home follow-up, supporting a favorable safety profile.

**What are the implications of the main findings?**

- Tramadol may represent a viable first-line alternative to conventional opioids for moderate procedural pain in spontaneously breathing EB neonates.
- These preliminary data support the need for multicenter studies to establish standardized analgesia protocols in this rare and vulnerable population.

**Abstract:**

**Background**: Inherited Epidermolysis Bullosa (EB) is a wide group of rare genetic disorders characterized by mucocutaneous fragility and blister formation. In neonates with EB, pain control is particularly complex because painful skin lesions coexist with developmental vulnerability, repeated handling, and the need for frequent wound care. Traditional opioid use carries a risk of adverse effects such as respiratory depression. Tramadol, a centrally acting weak opioid with a dual mechanism of action, may offer a safer alternative. **Methods**: This retrospective observational study analyzed neonates with different EB subtypes admitted to our tertiary neonatal care center between January 2020 and October 2022. Genetic diagnosis was confirmed via next-generation sequencing. Pain was assessed using the Neonatal Infant Pain Scale (NIPS). Tramadol was administered intravenously (1–2 mg/kg bolus or 0.1–0.2 mg/kg/h infusion) before daily wound dressings, then transitioned to oral dosing when appropriate. Pain scores before and after tramadol administration were compared. **Results**: Six neonates with various EB subtypes were included. All patients received tramadol for procedural pain control. No significant differences in NIPS scores were observed before and after tramadol administration (*p* = 0.997), indicating adequate pain control, although baseline pain scores were low, limiting interpretation of analgesic efficacy. No immediate adverse events were observed during hospitalization or reported during follow-up. **Conclusions**: Scheduled tramadol administration appears to be a safe and effective option for pain management in neonates with EB, with no observed hemodynamic or respiratory complications. Given the scarcity of data in this population, our findings highlight the need for multicenter studies to establish standardized analgesia protocols for EB neonates.

## 1. Introduction

The Inherited Epidermolysis Bullosa (EB) comprises a spectrum of inherited skin-fragility disorders in which minor mechanical trauma may cause blistering and erosions involving the skin and mucosal surfaces. There are four major types of EB: EB simplex (EBS), junctional EB (JEB), dystrophic EB (DEB), and kindler EB (KEB) [1]. In almost all EB types, newborns experience pain. The multiple mucocutaneous lesions are painful in rest conditions, with exacerbation after minimal procedures such as caregiving maneuvers and during dressings [2]. Pain in EB is often multifactorial and includes background pain, procedural pain during wound care, and breakthrough pain episodes, frequently requiring an individualized multimodal analgesic approach [3]. It is therefore mandatory to have an appropriate analgesia/sedation, as the devastating effects of non-controlled pain on newborns’ neurodevelopment are well known [4]. Neonatal pain management remains a significant challenge in clinical practice, especially in these patients [2,5], as the immature physiology of newborns complicates both the assessment and treatment of pain [6].

For mild pain, non-opioid analgesics (e.g., paracetamol) can be used to treat both acute and chronic cutaneous pain, whereas for moderate or severe pain, an opioid analgesic should be used [7].

Conventional opioids, while effective, are often limited by their adverse side effects and the risk of respiratory depression in this vulnerable population, particularly in EB neonates with involvement of the upper airways [6]. Tramadol provides analgesia through weak μ-opioid receptor activity and inhibition of serotonin and norepinephrine reuptake; however, its neonatal use requires caution because maturation-dependent clearance and CYP2D6 variability may influence both response and toxicity. Its unique pharmacokinetic and pharmacodynamic profile may offer a more balanced approach to pain control, potentially minimizing the risks associated with traditional opioid therapies [8].

The aim of this study was to describe our single-center experience with tramadol administration for procedural pain management during skin dressings in neonates with EB.

## 2. Materials and Methods

### 2.1. Study Design

In this retrospective observational study, we reviewed medical records of EB neonates admitted to the Neonatal Intensive Care Unit and the Neonatal Intermediate Care Unit of our reference center from January 2020 to October 2022. Medical history and clinical data were collected from the electronic medical records.

### 2.2. Genetic Analysis

EB diagnosis was established through clinical features, and, after signing a written informed consent by the parents for their children, a skin biopsy was performed for immunofluorescence antigen mapping and transmission electron microscopy, and then its results were confirmed by blood sampling for genetic testing. Genomic DNA was extracted from circulating leukocytes collected from the probands. Next-generation sequencing (NGS) was performed, using Twist Custom Panel kit—Clinical Exome (Twist Bioscience, San Francisco, CA, USA), on the NovaSeq6000 platform (Illumina Inc., San Diego, CA, USA). In silico analysis in trios (proband and parents) was performed for coding regions and exon–intron junctions of the genes associated with EB: LAMA3, LAMB3, LAMC2, COL7A1, ITGB4, ITGA6, COL17A1, KRT5, KRT14, EXPH5, KLHL24, PLEC, DST, PKP1 and FERMT1.

### 2.3. Clinical Management of EB and Pain Assessment

Clinical management was based on available international recommendations for neonatal EB care and wound management, emphasizing multidisciplinary care, atraumatic handling, parental involvement, and adequate analgesia during dressing procedures [7,9]. The dermatologists and the dedicated nurses taught the parents how to handle their child, break the blister roof, remove the crusts to reduce itching, and perform bathing and dressings [10]. Non-pharmacological pain management strategies included gentle handling, minimization of skin friction, parental involvement during care, and optimization of the dressing environment. The neonatologist was responsible for the pharmacological management of pain, feeding, and taking care of general conditions. The NIPS was employed for pain assessment, evaluating facial expression, cry, breathing patterns, arm movements, leg movements, and state of arousal [11]. The score can be interpreted as follows: (0–1) no pain; (2) mild pain; (3–4) moderate pain; and (5–7) severe pain [12]. Our general approach on an as-needed basis consisted of using paracetamol for mild pain and administering tramadol for moderate pain, given its efficacy and safety in achieving optimal pain control without compromising respiratory function. In case of severe pain, non-responsive to paracetamol and tramadol, we administered classical opioids (morphine or fentanyl) in case of severe pain.

Given the pain and stress related to daily dressings of EB patients, we used to give Tramadol 30 min before daily dressings on a scheduled basis rather than on an as-needed basis, and we compared NIPS values between pre-medication pain scores and post-medication pain scores. In this study, Tramadol was administered intravenously with boluses doses of 1–2 mg/kg or a continuous infusion of 0.1–0.2 mg/kg/h (0.1 mg/kg/h preterm newborn and 0.2 mg/kg/h in the full-term newborn) and then switched to the oral formulation as soon as possible (at the dose of 1–2 mg/kg).

### 2.4. Statistical Analysis

Data are presented as numbers and percentages for categorical variables. Continuous variables are expressed as mean ± standard deviation (SD) if they were normally distributed or as median and interquartile range if normality could not be accepted, according to the D’Agostino-Pearson test. Comparisons between pre-medication pain scores and post-medication pain scores were made with the Wilcoxon test. A *p*-value < 0.05 was considered significant. Data were analyzed with the MedCalc Software package for Windows, release 12.7 (MedCalc Software Ltd., Ostend, Belgium).

## 3. Results

### 3.1. Patients

Between January 2020 and October 2022, six neonates with EB were admitted to our neonatal units. The characteristics of these patients are shown in Table 1. All patients were initially admitted to the Neonatal Intensive Care Unit and then transferred to the Neonatal Intermediate Care Unit. The median age at admission was 9.5 days (IQR 2.3–22.8).

In four patients (66.7%), a central venous catheter (silicone Broviac catheter) (CVC) was placed. The median length of CVC stay was 32.0 days (IQR 26.3–37.3). In two patients, parenteral nutrition was required because of feeding difficulty related to painful blisters in the oral cavity, for a median length of 11.5 days (IQR 9.8–13.3). Only one patient (P4), affected with severe junctional EB, died in the hospital before discharge. A further patient with severe JEB (P3) died during the follow-up in the first year of life.

Three patients had a JEB, two had Recessive DEB (RDEB), and one had intermediate EBS with muscular dystrophy (Table 2).

### 3.2. Pain Management

Initially, five patients (83%) were treated with intravenous opioids (fentanyl or morphine) to manage severe pain for about 24–48 h on a scheduled basis; the remaining patient was regularly treated with oral morphine, given the less severe wounds, avoiding the positioning of a peripheral venous access.

Afterward, none of these patients received paracetamol, whereas all included patients were managed using tramadol. In particular, tramadol was administered intravenously in 5 patients (83%) for a median of 5.5 days (IQR 4.0–13.8) during hospital stay. Of them, only one (P4 with severe JEB) required a continuous infusion of tramadol for about 24 h before bolus administration.

We observed no differences in NIPS scores before daily medications (median value 0, IQR 0–1) and after (median value 0, IQR 0–0) (*p* = 0.997) when tramadol was used.

We observed no side effects related to tramadol administration (respiratory depression, hypotension, and bradycardia).

In all survivors, tramadol was continued at home in case of moderate pain, whereas the recommendation to use paracetamol was given in case of mild pain or less severe wounds: parents reported no adverse events during follow-up with this approach.

## 4. Discussion

Prolonged exposure to pain influences neonatal morbidity and has long-term effects [4,13]. Since midgestation, fetuses become capable of processing nociceptive stimuli, and brain development is continuously stimulated [14]. In particular, chronic stress and glucocorticoids disrupt neuronal integrity, altering glutamate signaling, impairing synaptic plasticity, and promoting neuronal apoptosis [15].

In the newborn, due to the wind-up phenomena, the release of mediators at the level of the spinal cord results in an increased sensitivity to pain in the adjacent dermatomes. Primary hyperalgesia occurs (i.e., increased sensitivity to painful stimuli); indeed, the threshold for pain perception is decreased, and even touch can transmit a painful sensation even after the painful stimulus has ended. Due to the phenomenon of so-called allodynia, the neonate perceives non-painful stimuli, such as touch, as painful. Repeated painful tissue damage in the neonatal period causes long-lasting proliferation of the dendritic endings of sensory nerves, and this hyperinnervation persists into adulthood [4,16,17].

This is crucial even more in EB newborns, where the intensity of pain is frequently related to disease severity and extent, and profoundly affects the quality of life of all family members [2,7].

The prevention of pain is considered more effective than the treatment of pain that is already ongoing. Therefore, the administration of analgesic drugs on an as-needed basis should be avoided, and instead, scheduled administration or continuous intravenous infusion should be used [18].

Tramadol is commonly classified as an opioid, albeit a “weak” mu agonist, with an affinity for mu receptors approximately 6000 times lower than morphine. It is thought to have the same potency as codeine and, as such, is routinely recommended by health care practitioners to avoid “stronger” opioids, such as morphine, with fewer adverse effects and lower respiratory depression [19].

In our EB newborns, we used the option of scheduled administrations, with the dual aim of delivering the minimum effective dose to avoid the risk of drug accumulation and achieving good pain control and preventing complications arising in the newborn from prolonged pain exposure. Pain should be regularly monitored, as a “fifth vital sign”, by recording the pain score along with the ongoing therapy in the medical record [20]. In this study, we compared NIPS values between pre-medication pain scores and post-medication pain scores, after a scheduled administration of tramadol, and we found no differences. These findings may suggest maintenance of low pain scores during dressing procedures; however, interpretation is limited by the low baseline NIPS values and the retrospective nature of the study. Obviously, we are aware that non-pharmacological treatments are cornerstones of neonatal pain management [21], and parental involvement is also fundamental to ensure optimal care and emotional support for these babies [22,23]. Parental presence and comforting interventions are increasingly recognized as important adjunctive strategies for procedural pain reduction in neonates [24].

The timing of medication administration before procedures is crucial and depends on the drug and the route of administration. Oral or intravenous non-steroidal anti-inflammatory drugs (NSAIDs), such as paracetamol, are effective in reducing the need for opioids in neonates [25]. These drugs should be administered as a first-line option, ideally 30–60 min prior to daily painful procedures [26]. This approach helps to manage pain effectively while minimizing the risks associated with opioid use. However, when daily procedures are more complex and prolonged, the use of opioids becomes necessary [26]. Oral tramadol has a precise onset of action (about 1 h after administration), while the onset of action of morphine orally administered is extremely variable, depending on the “first passage” effect [27].

Based on our clinical experience, tramadol appeared feasible and was not associated with observed immediate cardiorespiratory complications in this small cohort. Therefore, although the absence of observed adverse events in such a limited retrospective cohort cannot be considered definitive evidence of safety, we believe that it is important to report these findings because one of the major obstacles in advancing pain management strategies for EB neonates lies in the rarity of the condition, which makes conducting randomized controlled trials (RCTs) challenging.

Pain assessment results should also be interpreted cautiously. Baseline NIPS values were already low before medication administration, potentially reflecting a floor effect. Therefore, the absence of significant changes in pain scores cannot be interpreted as definitive evidence of analgesic efficacy or superiority. Furthermore, we must specify that, despite these advantages, it is essential to consider the limitations of tramadol use in neonates, especially in preterm infants. Tramadol pharmacokinetics in neonates remains highly variable because of developmental maturation processes affecting hepatic metabolism and renal elimination. In particular, CYP2D6 ontogeny and genetic polymorphisms may substantially influence both analgesic response and the risk of adverse events, resulting in marked interindividual variability during the first months of life [28,29]. Our EB patients were all full-term born.

Several international guidelines and expert consensus documents emphasize the need for anticipatory analgesia before wound care procedures in EB, although evidence regarding the optimal pharmacological strategy in neonates remains limited [30,31]. However, to the best of our knowledge, there are no specific experiences supporting its use with data, and this underscores the importance of sharing clinical experiences and observational data to refine and standardize pain management protocols for these vulnerable patients. This work seeks to contribute to the limited literature on pain management in this vulnerable population and to provide clinical insights that may help optimize treatment protocols for neonates with epidermolysis bullosa. In this context, our experience should be interpreted as preliminary observational evidence rather than as a recommendation for routine first-line tramadol use.

This study has several important limitations, including its retrospective single-center design, the very small sample size, the heterogeneity of EB subtypes, the absence of a comparator group, the lack of standardized long-term follow-up, and the intrinsic limitations of retrospective pain assessment. Therefore, our findings should be considered preliminary and hypothesis-generating.

## 5. Conclusions

In this small retrospective cohort, tramadol administration during daily wound dressings appeared feasible and was not associated with observed immediate respiratory or hemodynamic complications. Our findings suggest that tramadol may represent a potential analgesic option in selected neonates with EB, particularly when repeated painful procedures are required. However, given the exploratory nature of this study, the absence of a comparator group, and the limited sample size, no conclusions regarding superiority, routine first-line use, or definitive safety can be drawn.

Further multicenter randomized controlled trials are warranted to confirm these findings and evaluate long-term neurodevelopmental outcomes associated with early tramadol use in this neonatal population.

## Figures and Tables

**Table 1:** Characteristics of neonates with EB.

	*Patients (n = 6)*
Males, n (%)	3 (50%)
Gestational age (weeks)	39.5 (39.0–40.0)
Birthweight (grams)	2960 (2675–3243)
Birthweight Z-score, SDS	−1.21 (−1.4/−0.44)
Small for gestational age, n (%)	3 (50%)
Maternal age	29.5 (28.0–34.0)
Medically assisted reproduction techniques, n (%)	1 (17%)
Cesarean section, n (%)	1 (17%)
Apgar score at 5 min	9.5 (9.0–10.0)
In-hospital mortality before discharge, n (%)	1 (16.7%)

**Table 2:** EB subtype, genetic mutations, and disease extension in included patients.

	*EB Subtype*	*Involved Gene*	*Molecular Diagnosis*
**P1**	Intermediate RDEB	COL7A1	c.[352_353insCCCCCTT, 355_356insACA];[8245G>A] p.[Arg118ProfsTer13, Arg118_Thr119insAsn];[(Gly2749Arg)]
**P2**	Localized RDEB	COL7A1	c.[6751-1G>T];[3187C>T] p.[(?)];[(Gln1063Ter)]
**P3**	Severe JEB	LAMB3	c.[1978C>T];[1978C>T] p.[(Arg660*)];[(Arg660*)]
**P4**	Severe JEB	LAMB3	c.[31dupC];[31dupC] p.[(Leu11ProfsTer43)];[(Leu11ProfsTer43)]
**P5**	Intermediate JEB	COL7A1	c.[380-1G>A];[380-1G>A] p.[(?)];[?]
**P6**	Intermediate EBS with muscular dystrophy	PLEC	c.9403C>T; p.(Gln3135Ter) e c.5619_5620delGGinsC (p.Ala1874PfofsTer181).

The symbol “*” indicates a premature termination (stop) codon, predicting protein truncation and possible loss of function.

## Data Availability

The original contributions presented in this study are included in the article. Further inquiries can be directed to the corresponding author.

## Abbreviations

The following abbreviations are used in this manuscript:

CVC	Central venous catheter
DEB	Dystrophic epidermolysis bullosa
EB	Epidermolysis bullosa
EBS	Epidermolysis bullosa simplex
IQR	Interquartile range
JEB	Junctional epidermolysis bullosa
KEB	Kindler epidermolysis bullosa
NGS	Next-generation sequencing
NICU	Neonatal intensive care unit
NIPS	Neonatal Infant Pain Scale
NSAIDs	Non-steroidal anti-inflammatory drugs
RDEB	Recessive dystrophic epidermolysis bullosa
SD	Standard deviation

## References

[1] Has C., Bauer J.W., Bodemer C., Bölling M.C., Bruckner-Tuderman L., Diem A., Fine J.-D., Heagerty A., Hovnanian A., Marinkovich M.P. (2020). Consensus reclassification of inherited epidermolysis bullosa and other disorders with skin fragility. Br. J. Dermatol..

[2] Saad R., Duipmans J., Yerlett N., Plevey K., McCuaig C., Woolfe W., Steinau K., Phillips J., Azzopardi N., Thompson K. (2024). Neonatal epidermolysis bullosa: A clinical practice guideline. Br. J. Dermatol..

[3] Goldschneider K.R., Good J., Harrop E., Liossi C., Lynch-Jordan A., Martinez A.E., Maxwell L.G., Stanko-Lopp D. (2014). Dystrophic Epidermolysis Bullosa Research Association International (DEBRA International). Pain care for patients with epidermolysis bullosa: Best care practice guidelines. BMC Med..

[4] Williams M.D., Lascelles B.D.X. (2020). Early neonatal pain—A review of clinical and experimental implications on painful conditions later in life. Front. Pediatr..

[5] Abreu Molnar B., Levin L., Yun D., Morel K., Wiss K., Wieser J., Ward C., Trice H., Garcia-Romero M.T., Stephenson A. (2024). Inpatient management of epidermolysis bullosa: Consensus-based hands-on instructions for neonates and postneonates. J. Am. Acad. Dermatol..

[6] Hall R.W., Anand K.J.S. (2014). Pain management in newborns. Clin. Perinatol..

[7] Has C., El Hachem M., Bučková H., Fischer P., Friedová M., Greco C., Nevoránková P., Salavastru C., Mellerio J., Zambruno G. (2021). Practical management of epidermolysis bullosa: Consensus clinical position statement from the European Reference Network for Rare Skin Diseases. J. Eur. Acad. Dermatol. Venereol..

[8] Allegaert K., Rochette A., Veyckemans F. (2011). Developmental pharmacology of tramadol during infancy: Ontogeny, pharmacogenetics and elimination clearance. Paediatr. Anaesth..

[9] Pope E., Lara-Corrales I., Mellerio J., Martinez A., Schultz G., Burrell R., Goodman L., Coutts P., Wagner J., Allen U. (2012). A consensus approach to wound care in epidermolysis bullosa. J. Am. Acad. Dermatol..

[10] Retrosi C., Diociaiuti A., De Ranieri C., Corbeddu M., Carnevale C., Giancristoforo S., Marchili M.R., Salvatori G., degli Atti M.L.C., El Hachem M. (2022). Multidisciplinary care for patients with epidermolysis bullosa from birth to adolescence: Experience of one Italian reference center. Ital. J. Pediatr..

[11] Lawrence J., Alcock D., McGrath P., Kay J., MacMurray S.B., Dulberg C. (1993). The development of a tool to assess neonatal pain. Neonatal Netw..

[12] Sarkaria E., Gruszfeld D. (2022). Assessing neonatal pain with NIPS and COMFORT-B: Evaluation of NICU staff competences. Pain Res. Manag..

[13] Anand K.J., Hall R.W. (2007). Controversies in neonatal pain: An introduction. Semin. Perinatol..

[14] Mohamed S.H.M., Reissland N., Anand K.J.S. (2024). An evidence-based discussion of fetal pain and stress. Neonatology.

[15] Crochemore C., Lu J., Wu Y., Liposits Z., Sousa N., Holsboer F., Almeida O.F.X. (2005). Direct targeting of hippocampal neurons for apoptosis by glucocorticoids is reversible by mineralocorticoid receptor activation. Mol. Psychiatry.

[16] Hatfield L.A. (2014). Neonatal pain: What’s age got to do with it?. Surg. Neurol. Int..

[17] Van den Hoogen N.J., Patijn J., Tibboel D., Joosten E.A. (2020). Repetitive noxious stimuli during early development affect acute and long-term mechanical sensitivity in rats. Pediatr. Res..

[18] Batton D.G., Barrington K.J., Wallman C., Finley G.A. (2006). Prevention and management of pain in the neonate: An update. Pediatrics.

[19] Placencia J., Madden K. (2021). Pediatric palliative care pharmacy pearls—A focus on pain and sedation. Children.

[20] Gallo A.M. (2003). The fifth vital sign: Implementation of the Neonatal Infant Pain Scale. J. Obstet. Gynecol. Neonatal Nurs..

[21] Mangat A.K., Oei J.L., Chen K., Quah-Smith I., Schmölzer G.M. (2018). A review of non-pharmacological treatments for pain management in newborn infants. Children.

[22] Eissler A.B., Zwakhalen S., Stoffel L., Hahn S. (2022). Effectiveness of involving parents during painful interventions for preterm infants: A systematic review. J. Obstet. Gynecol. Neonatal Nurs..

[23] Ullsten A., Campbell-Yeo M., Eriksson M. (2024). Parent-led neonatal pain management: A narrative review. Front. Pain Res..

[24] Campbell-Yeo M., Dol J., Disher T., Benoit B., Chambers C.T., Sheffield K., Boates T., Harrison D., Hewitt B., Jangaard K. (2013). The Power of a Parent’s Touch: Evaluation of Reach and Impact of a Targeted Evidence-Based YouTube Video. J. Perinat. Neonatal Nurs..

[25] El Hachem M., Zambruno G., Bourdon-Lanoy E., Ciasulli A., Buisson C., Hadj-Rabia S., Diociaiuti A., Gouveia C.F., Hernández-Martín A., Laguna R.d.L. (2014). Multicentre consensus recommendations for skin care in inherited epidermolysis bullosa. Orphanet J. Rare Dis..

[26] Iohom G., Lyons B. (2001). Anaesthesia for children with epidermolysis bullosa: A review of 20 years’ experience. Eur. J. Anaesthesiol..

[27] Grond S., Sablotzki A. (2004). Clinical pharmacology of tramadol. Clin. Pharmacokinet..

[28] Allegaert K., Van den Anker J.N., De Hoon J.N., Van Schaik R.H.N., Debeer A., Tibboel D., Naulaers G., Anderson B. (2008). Covariates of tramadol disposition in the first months of life. Br. J. Anaesth..

[29] Allegaert K., Tibboel D., van den Anker J. (2013). Pharmacological treatment of neonatal pain: In search of a new equipoise. Semin. Fetal Neonatal Med..

[30] El Hachem M., Diociaiuti A., Bonamonte D., Brena M., Lospalluti L., Magnoni C., Neri I., Peris K., Tadini G., Zambruno G. (2025). Multidisciplinary Italian Delphi consensus for recessive dystrophic epidermolysis bullosa. Orphanet J. Rare Dis..

[31] Mellerio J.E., El Hachem M., Bellon N., Zambruno G., Bučková H., Autrata R., Salavastru C., Caldaro T., Greco C., Has C. (2020). Emergency management in epidermolysis bullosa: Consensus clinical recommendations. Orphanet J. Rare Dis..

